# Supplementary material for: Resolving the full spectrum of human genome variation using Linked-Reads
Source: Genome Res. 2019 Apr;29(4):635–45. doi: 10.1101/gr.234443.118 (PMC6442396; doi:10.1101/gr.234443.118)
Supplement: Supplemental Material [file supp_gr.234443.118_Supplemental_longranger-2.2.zip › longranger-2.2.tar.bz2!/lr2.2-public/lib/stan/cmdstan/stan/doc/api/html/check__corr__matrix_8hpp_source.html]

Stan: src/stan/math/error\_handling/matrix/check\_corr\_matrix.hpp Source File


|  |  |
| --- | --- |
|  | Stan  2.5.0  probability, sampling & optimization |


- Main Page
- Related Pages
- Namespaces
- Classes
- Files

- File List
- File Members

All Classes Namespaces Files Functions Variables Typedefs Enumerations Enumerator Friends Macros Pages

- src
- stan
- math
- error\_handling
- matrix

check\_corr\_matrix.hpp

Go to the documentation of this file.

1 #ifndef STAN\_\_MATH\_\_ERROR\_HANDLING\_\_MATRIX\_\_CHECK\_CORR\_MATRIX\_HPP

2 #define STAN\_\_MATH\_\_ERROR\_HANDLING\_\_MATRIX\_\_CHECK\_CORR\_MATRIX\_HPP

3

4 #include <sstream>

5

6 #include <stan/math/error\_handling/dom\_err.hpp>

7 #include <stan/math/error\_handling/check\_positive.hpp>

8 #include <stan/math/error\_handling/matrix/check\_pos\_definite.hpp>

9 #include <stan/math/error\_handling/matrix/check\_symmetric.hpp>

10 #include <stan/math/error\_handling/matrix/check\_size\_match.hpp>

11 #include <stan/math/error\_handling/matrix/constraint\_tolerance.hpp>

12 #include <stan/math/matrix/Eigen.hpp>

13 #include <stan/math/matrix/meta/index\_type.hpp>

14

15

16 namespace stan {

17

18  namespace math {

19

36  // FIXME: update warnings

37  template <typename T\_y, typename T\_result>

38  inline bool check\_corr\_matrix(const char\* function,

39  const Eigen::Matrix<T\_y,Eigen::Dynamic,Eigen::Dynamic>& y,

40  const char\* name,

41  T\_result\* result) {

42  using Eigen::Dynamic;

43  using Eigen::Matrix;

44  typedef typename index\_type<Matrix<T\_y,Dynamic,Dynamic> >::type size\_t;

45

46  stan::math::check\_size\_match(function,

47  y.rows(), "Rows of correlation matrix",

48  y.cols(), "columns of correlation matrix",

49  result);

50

51  stan::math::check\_positive(function, y.rows(), "rows", result);

52

53  stan::math::check\_symmetric(function, y, "y", result);

54

55  for (size\_t k = 0; k < y.rows(); ++k) {

56  if (!(fabs(y(k,k) - 1.0) <= CONSTRAINT\_TOLERANCE)) {

57  std::ostringstream message;

58  message << " is not a valid correlation matrix. "

59  << name << "(" << stan::error\_index::value + k

60  << "," << stan::error\_index::value + k

61  << ") is %1%, but should be near 1.0";

62  std::string msg(message.str());

63  return dom\_err(function,y(k,k),name,msg.c\_str(),"",result);

64  }

65  }

66  stan::math::check\_pos\_definite(function, y, "y", result);

67  return true;

68  }

69

70  }

71 }

72 #endif

check\_pos\_definite.hpp

dom\_err.hpp

stan::agrad::fabs

fvar< T > fabs(const fvar< T > &x)

**Definition:** fabs.hpp:18

stan::math::dom\_err

bool dom\_err(const char \*function, const T &y, const char \*name, const char \*error\_msg, const T\_msg error\_msg2, T\_result \*result)

**Definition:** dom\_err.hpp:36

check\_size\_match.hpp

check\_positive.hpp

stan::math::check\_size\_match

bool check\_size\_match(const char \*function, T\_size1 i, const char \*name\_i, T\_size2 j, const char \*name\_j, T\_result \*result)

**Definition:** check\_size\_match.hpp:13

constraint\_tolerance.hpp

check\_symmetric.hpp

stan::math::CONSTRAINT\_TOLERANCE

const double CONSTRAINT\_TOLERANCE

The tolerance for checking arithmetic bounds In rank and in simplexes.

**Definition:** constraint\_tolerance.hpp:11

stan::math::check\_symmetric

bool check\_symmetric(const char \*function, const Eigen::Matrix< T\_y, Eigen::Dynamic, Eigen::Dynamic > &y, const char \*name, T\_result \*result)

Return true if the specified matrix is symmetric.

**Definition:** check\_symmetric.hpp:31

stan::math::index\_type

Primary template class for the metaprogram to compute the index type of a container.

**Definition:** index\_type.hpp:21

stan::error\_index::value

**Definition:** traits.hpp:20

stan::math::check\_pos\_definite

bool check\_pos\_definite(const char \*function, const Eigen::Matrix< T\_y, Eigen::Dynamic, Eigen::Dynamic > &y, const char \*name, T\_result \*result)

Return true if the specified matrix is positive definite.

**Definition:** check\_pos\_definite.hpp:29

index\_type.hpp

stan::math::check\_positive

bool check\_positive(const char \*function, const T\_y &y, const char \*name, T\_result \*result)

**Definition:** check\_positive.hpp:54

stan::math::check\_corr\_matrix

bool check\_corr\_matrix(const char \*function, const Eigen::Matrix< T\_y, Eigen::Dynamic, Eigen::Dynamic > &y, const char \*name, T\_result \*result)

Return true if the specified matrix is a valid correlation matrix.

**Definition:** check\_corr\_matrix.hpp:38

Eigen.hpp


---

[ Stan Home Page ]

*© 2011–2014,
Stan Development Team.*
